# Social Cognitive Theory and Physical Activity: Examining Gender-Based Prediction Patterns and Theoretical Validity

**DOI:** 10.3390/sports13080249

**Published:** 2025-07-29

**Authors:** Viktoria Sophie Egele, Robin Stark

**Affiliations:** Department of Education, Saarland University, 66123 Saarbrücken, Germany; r.stark@mx.uni-saarland.de

**Keywords:** physical activity, validity, theoretical assumptions, gender differences

## Abstract

This study explored gender-specific nuances in the applicability of Social Cognitive Theory (SCT) to predict physical activity behavior. This study aimed to determine whether similar or different prediction patterns emerge for men and women, particularly emphasizing the tenability of the SCT model’s theoretical assumptions across gender. Six hundred fifty-four participants (58.1% women, 41.1% men) completed two validated questionnaires at separate time points (t1 = social cognitive and demographic variables; t2 = physical activity behavior). We employed a multigroup Structural Equation Model (SEM) to examine the validity of the theoretical assumptions and the influence of gender. The results suggest that SCT’s theoretical assumptions hold true for men and women, indicated by a highly satisfactory fit of the SEM despite the variance explained being small (R^2^_women_ = 11.9%, R^2^_men_ = 7.3%). However, the importance of the specific theoretical paths and the underlying mechanisms of action might differ between genders, and the interplay of the social and cognitive variables to predict physical activity may vary significantly for men and women. The use of SCT can be recommended for explaining and predicting physical activity behavior, although gender-specific differences in the underlying theoretical relationships should be taken into consideration when designing interventions or when being used to explain physical activity behavior.

## 1. Introduction

**Figure 1:**
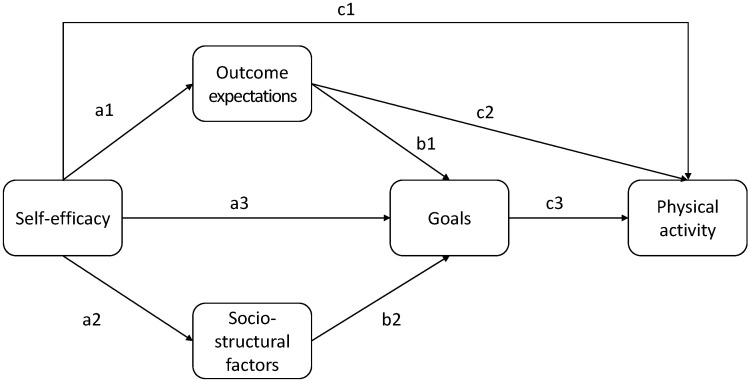
Social Cognitive Theory. This figure was previously published by Egele et al. [3], as proposed by Bandura [4]. Note: The nomenclature of these paths (a1, a2, a3, b1, b2, c1, c2, c3) is entirely subjective, arising from the authors’ personal predilection for the conceptualization of the SEM.

Albert Bandura’s social cognitive theory (SCT) is among the most prominent psychological theories on human behavior [1]. It is notable for its comprehensive scope, encompassing almost the entire field of psychological factors. The theory posits that personal, behavioral, and environmental factors interact to influence an individual’s behaviors, facilitating a holistic examination of the socio-cognitive factors associated with human action. SCT postulates that a dynamic interplay between these factors shapes individual behaviors. The theory places a particular emphasis on self-efficacy (the confidence in the ability to successfully perform physical activity, even when facing obstacles) and outcome expectations (expectations concerning the potential positive and negative consequences of being physically active). The theory also encompasses goal setting (the establishment of explicit, attainable physical activity objectives) and sociostructural factors (environmental elements such as facility availability, social support, and time constraints) [2]. According to the theory, self-efficacy is the most influential predictor of physical activity behavior. A positive linkage has been demonstrated between high self-efficacy and expectations of positive outcomes, the establishment of more concrete goals, and an increased level of participation. Individuals who possess high self-efficacy demonstrate a propensity to persist in physical activity despite the presence of adversity. Figure 1 illustrates the theoretical model.

A substantial body of literature has tested and confirmed several aspects of SCT in relation to behavior in both specific populations and selected domains, as well as in the context of physical activity behavior [3,5,6,7,8,9].

However, SCT has rarely been subjected to comprehensive testing and modeling following Bandura’s original intentions. Studies have often only examined selected elements; this was critiqued by Young et al. [9] in a review of studies on SCT pertaining to physical activity, with the authors concluding that “the majority of SCT research has focused solely upon self-efficacy or examined self-efficacy in combination with only one or two other variables” (p. 985). Thus, it would appear to be important to examine the theory as Bandura originally intended, including all four constructs with their assumed direct, indirect, and total effects. Moreover, evidence supporting the power of SCT to predict and explain physical activity has primarily been demonstrated for specific samples, such as those with diseases like multiple sclerosis (e.g., [6,10]), type 2 diabetes (e.g., [8]), and severe mental illness (e.g., [11]). The theory has also been applied to understand the experiences of individuals with cancer (e.g., [5,12]) and those dealing with obesity (e.g., [13]), as well as Crohn’s disease [14]. The existing literature on SCT in regard to physical activity behavior concerning healthy adult populations has been relatively sparse [3,9,13].

Although a more nuanced comprehension of the behavioral antecedents in these particular samples is undoubtedly a point of interest, it is imperative to acknowledge that the phenomenon of physical inactivity is not exclusive to those samples. As evidenced by Guthold’s [15] and Strain’s [16] comprehensive reviews, physical inactivity is a pervasive global issue. The studies revealed that approximately 30% of adults globally fail to attain the physical activity level recommended by the World Health Organization (WHO) [16] for adults to be at least 150 min moderately physically active or 75 min vigorously physically active per week. The objective of these recommendations is to promote substantial health benefits [17].

It can be reasonably deduced that investigating SCT with a focus on a holistic model test in a large population of healthy individuals may be of significant interest. Promising results have emerged from other recent studies with objectives aligned with those of the present study, leading to the assumption that the theoretical assumptions of the model in question satisfactorily reflect reality.

However, prior research findings concerning physical activity and SCT elements have also indicated the existence of notable gender disparities, and the importance of a gender-sensitive approach is currently being discussed [18]. A substantial body of literature has documented the existence of gender differences in self-efficacy related to physical activity, with men having stronger self-efficacy beliefs than women [19,20,21] and self-efficacy being differently related to behavior for men than for women [21]. Anderson et al. [22] documented differences in the outcome expectations of men and women. Additionally, studies have indicated that the correlations between outcome expectations and behavior are gender specific. For instance, Trost et al. [23] demonstrated that outcome expectations were a significant predictor of physical activity behavior for men but not for women. In regard to sociostructural factors, a definitive conclusion concerning gender differences remains elusive, although some research has indicated that barriers may impact women more than men [24]. The contradictory findings observed in this domain may be contingent upon the conceptualization employed [25,26]. Similarly, the existing evidence on gender-specific objectives is not yet sufficiently clear to permit drawing definitive conclusions. Conversely, clear gender differences have been demonstrated for physical activity, with studies consistently demonstrating variations in men’s and women’s participation, intensity, level, and preferred types of exercise [27,28]. Not only do men report and demonstrate higher physical activity levels than women, but they also report engaging in a higher intensity of physical activity. Such differences remain largely consistent throughout the lifespan [16,23,29,30,31,32].

Therefore, alongside the general validity of the theoretical assumptions, their universality should also be tested, namely that the model’s theoretical assumptions are tenable across gender and that the theory can be used equally effectively for men and women to explain and predict physical activity behavior.

Drawing on recent research results [3], we hypothesize that SCT—when modeled as Bandura proposed—fits the data and describes the theoretically stated relations between self-efficacy, outcome expectations, sociostructural factors, goal setting, and physical activity in its entirety (H1). We thereby aim to replicate the hypothesis of Egele et al. [3]. Furthermore, given the gender differences reported for multiple SCT elements [21,23,24], we investigate the model fit individually for men and women using a multigroup Structural Equation Model (SEM) (H2). We assume that the theoretically stated relations between self-efficacy, outcome expectations, sociostructural factors, goal setting, and physical activity are valid for men and women separately and explore potential differences in the prediction patterns.

## 2. Materials and Methods

### 2.1. Participants

Several methods were used to recruit participants, including the placement of notices on the campus of Saarland University and other cultural centers in Germany and online advertisements. Prior to analysis, we excluded data from any subjects who did not complete both questionnaires (n = 36). In addition, the data were checked for response time and response pattern usage, which led to the exclusion of two more participants. The final sample comprised 654 German participants (58.9% women, 41.1% men) aged between 18 and 68 (M = 31.90, SD = 13.18). The mean age of the 385 women was 30.40 years (SD = 12.66), while the mean age of the 269 men was 34.04 years (SD = 13.66). Participants reported an average of 2685.44 metabolic equivalent of task (MET) minutes of total physical activity per week (SD = 2630.32). This amount was comparable to the physical activity reported in similar studies [3,30], yet clearly exceeded the WHO’s recommended physical activity levels, which are equivalent to approximately 600 MET minutes [17].

### 2.2. Instruments

The scales for measuring the SCT components were taken from a validated German scale [33], allowing a specific evaluation of the components of SCT within the context of physical activity. The scales comprise 18 items in total and have demonstrated adequate psychometric properties [33]. Five items measured self-efficacy, and the subscale’s reliability was α = 0.912 (McDonald’s Omega = 0.904). Five items were used to assess outcome expectations, and this subscale’s reliability was α = 0.906 (McDonald’s Omega = 0.801). The subscale for measuring goals consisted of four items, and its reliability was α = 0.889 (McDonald’s Omega = 0.884). Finally, sociostructural factors were assessed by four items, with the subscale’s reliability at α = 0.751 (McDonald’s Omega = 0.604).

Physical activity was measured using seven items of the International Physical Activity Questionnaire—Short Form in German (IPAQ-SF, [34]), which assesses general physical activity over the previous seven days. The questionnaire uses an open response format to assess the number of days and the average time (hours and minutes) spent on physical activity and sitting. We selected the IPAQ-SF because of its parsimony, good psychometric qualities, and implementation in multiple existing studies [35,36,37]. According to the scoring protocol, the MET as a measure of physical activity was calculated as a weighted sum of vigorous and moderate physical activity and time spent walking. The resulting score was divided by 1000 to obtain scores of comparable ranges for SCT and physical activity.

Demographics, gender, and age were assessed using open questions.

### 2.3. Procedure

This study was conducted in compliance with the ethical standards of the Declaration of Helsinki and was approved by the Ethics Committee of the Faculty of Empirical Human and Economic Sciences of Saarland University (Approval 24–32) on 18 November 2024. All subjects provided written informed consent prior to data collection. All hypotheses were specified before data collection.

This study was conducted entirely online, with the questionnaires implemented using SoSci Survey (Version 3.7.06) [38]. In the first questionnaire, the participants answered the scales to assess the SCT components and provided demographic information. They provided information on their physical activity one week later.

### 2.4. Data Analyses

R (Version 4.4.0, R Core Team, 2020, Vienna, Austria), specifically the packages Lavaan [39] and psych [40], was used for the calculations. The model depicted in Figure 1 was set up as an SEM with latent variables, and physical activity was included as a manifest variable. We implemented the MLM estimator with the Satorra–Bentler-scaled χ^2^-test statistic and robust standard errors, as was also carried out previously in similar studies [3], since some variables exhibited a skewed distribution. We included the indirect and total effects through defined parameters.

To test Hypothesis 1, we considered the model’s fit in the whole sample. The first step examined each latent variable’s measurement model with a single-factor confirmatory factor analysis (CFA). The second step included a CFA with all four latent variables to evaluate the entire measurement model’s fit. The fit of the model was evaluated following Schermelleh-Engel et al.’s [41] criteria: the χ^2^-test statistic should be greater than *p* = 0.05, RMSEA should be smaller than 0.05 with a *p*-value greater than 0.10 for the test of close fit, CFI should be greater than 0.97, and SRMR should be smaller than 0.05. In the third step, the SEM was estimated and evaluated against the above criteria, hereafter called SEM_1_. Standard fit measures of the SEM do not allow an assessment of the theoretically stated relations only in the structural part, since they simultaneously assess both the fit of the measurement and the structural parts. As such, we followed Lance et al.’s [42] recommendations; Lance et al. [42] recommended looking at the C9 and C10 indexes to evaluate the structural model. As also described in our prior study [3], the C9 index is an index of goodness-of-fit, ranging from 0 to 1. A value closer to 1 indicates a better fit of the structural model. C9 reflects a model comparison of the theoretical model and a model with no relations between the latent variables. The C10 index is a badness-of-fit index, also ranging between 0 and 1. A value closer to 0 indicates a better fit of the structural model. C10 reflects a model comparison of the theoretical model and a model with no assumed paths between latent variables. We used formulas based on the models’ χ^2^-test statistics, as provided by Lance et al. [42], to calculate C9 and C10, hereafter called C9_1_ and C10_1_. We modeled a CFA containing the covariances of the latent variables with MET to estimate the structural model part only with the traditional fit indices.

We calculated and tested the direct, indirect, and total effects with a nominal significance level of α = 0.05.

To test Hypothesis 2, we first tested the measurement invariance between men and women. Following the recommendations of Guenole et al. [43] for comparing the regression coefficients, the measurement invariance up to the level of metric measurement invariance was established. After establishing measurement invariance across groups, we ran a multigroup SEM (hereafter referred to as SEM_2_) and employed a Wald test to test for overall group differences by simultaneously comparing all direct paths across the two groups.

Fit measures for the two groups were evaluated against Schermelleh-Engel et al.’s [41] criteria. To assess the validity of the theoretically stated relations for men and women separately, we evaluated C9 and C10 indices (C9_2M_, C9_2W_, C10_2M_, and C10_2W_). Two CFAs were modeled, containing the covariances of the latent variables with MET (hereafter referred to as partial structural model_2M_ and partial structural model_2W_) to assess the structural model part only with traditional fit indices.

Again, the direct, indirect, and total effects for men and women separately were calculated and tested, with a nominal significance level of α = 0.05.

Data are available on request.

## 3. Results

Table 1 displays the single-factor measurement models and the CFA with all four latent variables, demonstrating a satisfactory model fit for each model.

For Hypothesis 1, the SEM fit was good, as Table 1 reports. The partial SEM, in which the structural part was separated from the measurement model, also indicated a good fit. The C9 and C10 indices were 0.988 and 0.012, respectively, which indicated a very good fit for the structural model part. The results support Hypothesis 1, confirming that the theoretical assumptions of SCT were valid in the overall sample. Table 1 depicts the direct, indirect, and total effects of the overall model.

For Hypothesis 2, we first established configural invariance to test measurement invariance. The fit indices of the model were close to Schermelleh-Engel et al.’s [41] criteria: χ^2^ (248) = 385.275, *p* < 0.001, CFI = 0.972, RMSEA = 0.045, 90% CI [0.036–0.054], *p*_close_ = 0.806, SRMR = 0.061. Therefore, we assumed configural invariance as given. Then, the factor loadings were constrained to be equal across groups to test metric invariance. The model fit was good: χ^2^ (262) = 398.494, *p* < 0.001, CFI = 0.972, RMSEA = 0.044, 90% CI [0.033–0.047], *p*_close_ = 0.875, SRMR = 0.064. Comparing the two models’ fit, the difference in χ^2^ was not significant: χ^2^ (14) = 13.14, *p* = 0.515, and the change in CFI was ΔCFI < 0.001. Based on Meade et al.’s [44] suggested threshold of ΔCFI = 0.002 and the non-significant difference in χ^2^, we concluded that metric invariance was supported by our data.

We then examined the model fit of the multigroup SEM; the model fit was good, as depicted in Table 1. For men and women separately, the fit of the structural part was very good. The C9_M_ index was 0.997, the C9_W_ index was 0.975, the C10_M_ index was 0.003, and the C10_W_ index was 0.025. Both partial SEMs also indicated a good fit for the structural models.

**Figure 2:**
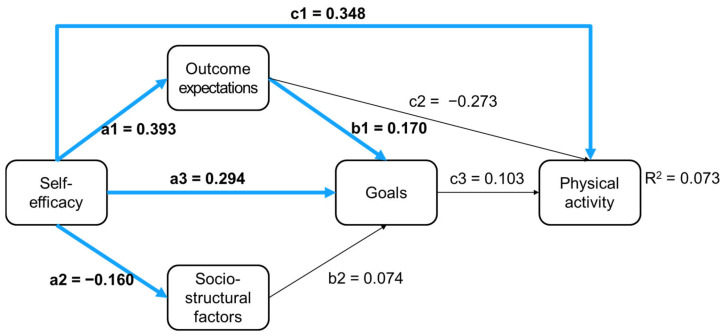
Direct paths in SCT for men; significant paths are marked in blue.

**Figure 3:**
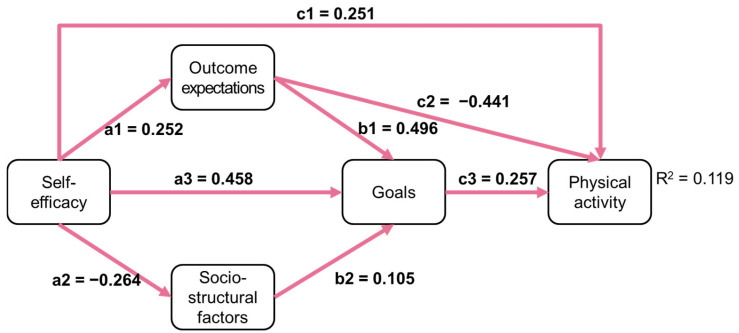
Direct paths in SCT for women; significant paths are marked in red.

A comparison of all direct paths of SCT across the two groups indicated no significant differences, Wald (8) = 12.718, *p* = 0.122. Given the satisfactory fit of the structural model for men and women and since there were no significant differences in the Wald test, we concluded that SCT’s theoretically stated relations appear to hold for both men and women. However, as Table 2 depicts, some paths appear to be only significant for women: sociostructural factors on goals (b2), outcome expectations on goals (c2), and goals on physical activity (c3). Figure 2 and Figure 3 illustrate the direct paths for men and women, with significant paths marked in blue and red, respectively.

## 4. Discussion

This study aimed to replicate the validity of the theoretical assumptions of SCT and investigate whether these assumptions are tenable for both men and women.

In this section, we will first briefly discuss the findings of the replication of Egele et al. [3] and then discuss the applicability and tenability of the theoretical assumptions of SCT to men and women.

In contrast to the findings of Egele et al. [3], the present study found that all hypothesized direct and indirect paths were statistically significant. This outcome is aligned with the predictions of Bandura’s theoretical framework. Only two pathways were not significant in the present study: the indirect effect of self-efficacy via sociostructural factors on goals and the indirect path of self-efficacy via sociostructural factors and goals on behavior. The underlying reasons for this phenomenon are likely attributable to the pathways’ intricate nature. They will be further elucidated later, as the pathways will be examined independently for men and women.

Thus, the findings of the present study indicated that a greater number of paths were significant compared to those of Egele et al. [3]. This discrepancy may be attributed to the sample size and its associated statistical power. In the present study, the number of participants exceeded three times the number of subjects in Egele et al.’s [3] study. Nonetheless, both studies support the fundamental assumption that SCT’s theoretical assumptions appear valid.

Furthermore, the findings of the present study confirm that the theoretical assumptions of SCT appear valid and applicable to both men and women. However, it appears that some paths may be more pertinent for women than men. Self-efficacy and outcome expectations appear to be of importance for both men and women. However, it appears that for men, the model’s complexity may be reduced to a direct path of self-efficacy on behavior, which is seemingly of great importance, while other paths seem less relevant. For instance, the influence of sociostructural factors on goals seems to be a notable phenomenon for women but not for men. Similarly, the path from outcome expectations to goals was significant for women, but not for men, as was the path from goals to behavior. Thus, for men, it appears that only self-efficacy plays a role in physical activity behavior, while for women, the complex path network seems to be more appropriate. This raises the question of what the underlying causes of these observed differences may be and invites a closer look.

It seems that men may possess greater self-efficacy than women; at least, this is suggested by the literature demonstrating that men have stronger self-efficacy beliefs than women, both generally and in relation to physical activity [19,20,21]. This is further supported by recent findings on adolescent populations, which have revealed strong gender-specific emotional and behavioral profiles that influence physical activity levels [45]. It is similarly conceivable that the assessment of SCT constructs itself exerts a somewhat limiting effect in this regard. For instance, it may be that women are more likely to have physical outcome expectations concerning the outcomes in question, which include health, good looks, and fitness, while men are more likely to have affective outcome expectations, such as enjoyment of sport and exercise [46]. However, contradictory research results have also been reported [47], indicating the necessity of clarification in future research. The scale employed in this study predominantly inquired about physical, rather than affective, outcome expectations [33], potentially contributing to the observed discrepancy in the outcome expectations guiding action between men and women. To obtain a more nuanced understanding of gender differences, it may be necessary to employ more differentiated data collection instruments, as was carried out by Gellert et al. [48]. This could also help ascertain whether self-efficacy expectations are indeed the primary motivating factor for men’s actions. The argument that a more differentiated assessment may be beneficial is further supported by the apparent validity of the theoretical model’s assumptions regarding the complex interplay of social and cognitive factors for men. While the present study could not provide definitive support that these complex pathways guide physical activity in men, the more differentiated recording of SCT components appears to open a meaningful new research avenue. Given the rarity of comprehensive testing of the theory and the lack of separate examinations of men and women [9], it would be beneficial to enhance their efficacy by pursuing this line of research further, particularly in the context of Social Cognitive Theory-based interventions. If it is indeed determined that self-efficacy is the primary determinant of behavior for men, whereas it is a multi-faceted interplay of social and cognitive influences for women, then adaptations to the intervention may be valuable.

Furthermore, the examination of the variance in physical activity that can be ascribed to social and cognitive variables merits closer scrutiny. Comparing the current study with others on SCT in the context of physical activity reveals a low variance level explained in the present study. Overall, 10% of the variance in physical activity was explained by SCT; social cognitive variables explained 7% of the variance in physical activity for men and 12% for women. The discrepancy between the intended and actual variance explained can be partially attributed to the intention–behavior gap. The discrepancy between individuals’ self-set goals and actual behaviors has been extensively documented in prior research, as exemplified by Feil et al. [49] in the context of physical activity. This was also evident in the current study, wherein the explained variance in goals was significantly higher overall (31%) as well as for men (27%) and women (37%) than in behavior. In meta-analyses (e.g., [49]), the correlation between goals and behavior has been observed to be at the random level, indicating that the issue of limited variance explained in the criterion may not be a limitation of the present study but, rather, a phenomenon warranting consideration within a broader context.

However, with this little share of variance explained, the theory does not meet Baranowski et al.’s [50] recommendations of R^2^ ≥ 0.30 to be considered a useful framework for intervention designs. The question thus arises as to why less variance could be explained in the current study than in prior ones.

This may also be attributed to our conceptualization of the construct of “physical activity.” Ours was a relatively broad conceptualization, as is common practice in many population-based surveys (e.g., [15,16]), to encompass as many individuals and diverse exercise behaviors as possible. SCT may explain a greater proportion of the variance if the behavior in question were more clearly defined. For instance, if physical activity was operationalized as a weekly frequency of ≥30 min exercise sessions and SCT scales were adapted to this criterion, there might be a higher correspondence and, subsequently, a stronger relationship. Consequently, we recommend that this study be replicated with an alternative physical activity criterion to further elucidate the underlying mechanisms.

In light of this study’s findings, it remains debatable whether SCT is a suitable basis for interventions, since the model’s theoretical assumptions are valid for both men and women and, thus, are both tenable and applicable to a diverse demographic. Conversely, the low variance explained may require a reconsideration of the unreserved recommendation of the theory.

### Limitations

One potential limitation of this study is the recording of the constructs, including social cognitive variables and physical activity behavior. This study replicated the procedure established by Egele et al. [3] and employed the same study design and questionnaires. However, there are several points worthy of further discussion.

In terms of the recording of the social cognitive variables, it is noteworthy that we utilized a validated questionnaire to assess SCT. This approach is more rigorous than that employed in many studies using non-validated questionnaires, a practice that has drawn critique from Young et al. [9], among others. However, the questionnaires are economic yet relatively brief, precluding the formulation of more precise statements about construct elements, including, for example, different types of outcome expectations. It might be conceivable that specific facets demonstrate gender-based differences; however, the questionnaire employed cannot ascertain this. The incorporation of additional facets of the SCT constructs is also conceivable concerning sociostructural factors, such as social support from family and friends, living conditions, and governmental support. In regard to objectives, assessing goal strength and specificity may be of additional value.

Furthermore, in regard to the recording of physical activity behavior, the assessment employed is open to criticism. Given the lack of consensus regarding the optimal method for recording physical activity behavior [51], our approach was guided by larger population comparison studies (e.g., [15,16]). Young et al.’s [9] meta-analysis also demonstrated that the assessment of physical activity behavior, subjectively or objectively, does not significantly impact the explained variance. However, it is essential to note that the recording of physical activity behavior through retrospective self-reports can be susceptible to potential biases and may not reach the desired validity, as previously evidenced by Egele et al. [52] and Straßburg et al. [53]. More specifically, social desirability [52] and recall bias [54] may result in bias in self-reported health behaviors. Thus, future studies might benefit from the inclusion of more objective assessment methods, such as accelerometers.

A further limitation of this study is the temporal spacing of the two data collection points. While two data collection points are already a step forward from the simultaneous assessment of the elements of SCT and physical activity—as previously conducted in a number of studies [9]—the evaluation of the elements of SCT and physical activity one week apart may still have restricted causal inference. Future studies may benefit from implementing a more longitudinal approach to enable a more thorough examination of causal inference.

## 5. Conclusions

The objective of this study was to assess the applicability of SCT for men and women and determine whether a similar or different gender-specific prediction pattern emerges. The results indicate that the theoretical assumptions appear to be valid for both the overall sample and for men and women separately. Consequently, the use of SCT can be recommended in the context of physical activity; however, potential gender-specific differences in the underlying theoretical relations should be considered, and the low variance explained may require a reconsideration of the unreserved recommendation of the theory. The present study is, thereby, positioned as basic research with the purpose of investigating causal relationships between SCT elements in relation to general physical activity. Pursuant to this study’s findings, which are partly exploratory in nature, subsequent research endeavors may concentrate on implementing the results within the domain of public health interventions to address the issue of physical inactivity. According to the findings of this study—though partially exploratory—it may be appropriate for men to primarily concentrate on sources of self-efficacy in order to increase self-efficacy, while a multi-perspective approach may be more appropriate for women. This approach would involve targeting sources of self-efficacy, modifying outcome expectations, and considering sociostructural factors and goals.

## Figures and Tables

**Table 1:** Fit measure of the measurement and Structural Equation Models.

	χ^2^	*df*	*p*	CFI	SRMR	RMSEA [CI]	*p*_close_
Self-efficacy	6.734	3	0.081	0.998	0.009	0.052 [0.000–0.106]	0.546
Goals	6.051	2	0.049	0.997	0.013	0.056 [0.005–0.126]	0.265
Outcome expectations	7.266	3	0.064	0.993	0.017	0.054 [0.000–0.106]	0.365
Sociostructural factors	1.104	1	0.296	1.000	0.008	0.013 [0.000–0.111]	0.577
Complete CFA	230.202	124	<0.001	0.978	0.053	0.040 [0.032–0.048]	0.979
Hypothesis 1							
SEM_1_	258.545	140	<0.001	0.976	0.052	0.039 [0.032–0.047]	0.991
Null modell_1_ *	735.883	-	-	-	-	-	-
All paths SEM_1_ *	369.726	-	-	-	-	-	-
Partial structural model_1_	<0.001	2	<0.001	1.000	<0.001	0.000 [0.000–0.000]	>0.999
Hypothesis 2							
SEM_2overall_	444.044	294	<0.001	0.970	0.062	0.043 [0.035–0.051]	0.022
SEM_2M_	210.152	-	-	-	-	-	-
SEM_2W_	233.892	-	-	-	-	-	-
Null modell_2M_ *	395.332	-	-	-	-	-	-
Null modell_2W_ *	450.108	-	-	-	-	-	-
All paths SEM_2M_ *	209.602	-	-	-	-	-	-
All paths SEM_2W_ *	228.396	-	-	-	-	-	-
Partial structural model_2M_	<0.001	2	<0.001	1.000	<0.001	0.000 [0.000–0.000]	>0.999
Partial structural model_2W_	<0.001	2	<0.001	1.000	<0.001	0.000 [0.000–0.000]	>0.999

Note: * C9 and C10 indices were calculated by the null and all paths SEMs. This table only reports χ^2^-test statistics, as the models were not judged in regard to their model fit.

**Table 2:** Direct, indirect, and total effects for the standardized regression coefficients both overall and for men and women separately.

Paths		Estimate	*SE*	*z*	*p*
Total effect					
Total effect	Overall	0.336	0.073	4.595	<0.001
	Men	0.338	0.152	2.227	0.026
	Women	0.327	0.075	4.383	<0.001
Direct effects					
a1 Self-efficacy → Outcome expectations	Overall	0.311	0.038	8.223	<0.001
	Men	0.393	0.058	6.724	<0.001
	Women	0.252	0.037	6.745	<0.001
a2 Self-efficacy → Sociostructural factors	Overall	−0.219	0.047	−4.657	<0.001
	Men	−0.160	0.067	−2.381	0.017
	Women	−0.264	0.059	−4.441	<0.001
a3 Self-efficacy → Goals	Overall	0.396	0.059	6.695	<0.001
	Men	0.294	0.101	2.920	0.004
	Women	0.458	0.069	6.646	<0.001
b1 Outcome expectations → Goals	Overall	0.504	0.111	4.543	<0.001
	Men	0.578	0.170	3.392	0.001
	Women	0.496	0.139	3.573	<0.001
b2 Sociostructural factors → Goals	Overall	0.102	0.043	2.350	0.019
	Men	0.074	0.074	0.996	0.319
	Women	0.105	0.053	1.978	0.048
c1 Self-efficacy → Physical activity	Overall	0.307	0.077	3.975	<0.001
	Men	0.348	0.149	2.340	0.019
	Women	0.251	0.089	2.832	0.005
c2 Outcome expectations → Physical activity	Overall	−0.355	0.137	−2.596	0.009
	Men	−0.273	0.227	−1.204	0.229
	Women	−0.411	0.170	−2.417	0.016
c3 Goals → Physical activity	Overall	0.183	0.055	3.351	0.001
	Men	0.103	0.065	1.591	0.112
	Women	0.257	0.082	3.154	0.002
Indirect effects					
a1 × c2 Self-efficacy → Outcome expectations → Physical activity	Overall	−0.068	0.017	−4.053	<0.001
	Men	−0.063	0.028	−2.232	0.026
	Women	−0.066	0.018	−3.673	<0.001
a1 × b1 Self-efficacy → Outcome expectations → Goals	Overall	0.157	0.037	4.215	<0.001
	Men	0.227	0.073	3.126	0.002
	Women	0.125	0.038	3.293	0.001
a1 × b1 × c3 Self-efficacy → Outcome expectations → Goals → Physical activity	Overall	−0.029	0.012	2.422	0.015
	Men	0.023	0.019	1.266	0.206
	Women	0.032	0.015	2.209	0.027
a3 × c3 Self-efficacy → Goals → Physical activity	Overall	0.072	0.022	3.284	0.001
	Men	0.030	0.019	1.593	0.111
	Women	0.118	0.039	3.027	0.002
a2 × b2 Self-efficacy → Sociostructural factors → Goals	Overall	−0.022	0.011	−2.107	0.034
	Men	−0.012	0.013	−0.943	0.346
	Women	−0.028	0.015	−1.792	0.073
a2 × b2 × c3 Self-efficacy → Sociostructural factors → Goals → Physical activity	Overall	−0.004	0.002	−1.936	0.053
	Men	−0.001	0.001	−0.841	0.400
	Women	−0.007	0.004	−1.727	0.084
b1 × c3 Outcome expectations → Goals → Physical activity	Overall	−0.092	0.038	2.457	0.014
	Men	0.060	0.047	1.275	0.202
	Women	0.128	0.055	2.309	0.021
b2 × c3 Sociostructural factors → Goals → Physical activity	Overall	−0.019	0.009	0.037	0.037
	Men	0.008	0.009	0.859	0.390
	Women	0.027	0.014	1.875	0.061
a1 × c2 + a1 × b1 + c3 + a2 × b2 × c3 + a3 × c3 Complete indirect path of self-efficacy	Overall	0.029	0.034	0.839	0.401
	Men	−0.010	0.045	−0.224	0.822
	Women	0.076	0.050	1.543	0.123

Note: Figure 1 displays the designation of the path coefficients.

## Data Availability

Data are available from the authors upon request. The data are not publicly available due to privacy and ethical restrictions.

## Abbreviations

The following abbreviations are used in this manuscript:

SCT	Social Cognitive Theory
SEM	Structural Equation Model
